# O051: European medical students and antibiotic stewardship: a multicentre survey of knowledge, attitudes and beliefs

**DOI:** 10.1186/2047-2994-2-S1-O51

**Published:** 2013-06-20

**Authors:** OJ Dyar, C Pulcini, P Howard, D Nathwani

**Affiliations:** 1 Oxford University, Oxford, UK; 2 Centre Hospitalier Universitaire de Nice, Nice, France; 3 Leeds University, Leeds, UK; 4 Dundee University, Dundee, UK

## Objectives

To learn about medical students’ knowledge and perspectives on antibiotic stewardship.

## Methods

Final year students at seven European medical schools (Dundee, Geneva, Linköping, Ljubljana, Madrid, Nice, Oxford) were invited to participate in an anonymous online survey in June 2012. Descriptive statistics are presented here.

## Results

The response rate was 35% (322/961). Regarding prescribing according to stewardship principles, students at all medical schools felt most confident in diagnosing infections and choosing the right antibiotic, and least confident in choosing combination therapies, and making the decision to not prescribe antibiotics in cases of diagnostic uncertainty.

With respect to the success of stewardship efforts thus far, the majority of students (83%) incorrectly thought MRSA bacteraemia rates had significantly increased over the past decade in their countries, and a quarter of students thought that handwashing was not at all an important contributor to resistance.

Most students (66%) thought the antibiotics they will prescribe will contribute to resistance, with almost all (98%) acknowledging that resistance will be a greater problem in the future. Students were aware that around 30% of antibiotic usage was unnecessary or inappropriate, with 83% feeling that such prescribing is unethical. Only 65% of students had been shown how to access their hospital’s guidelines. As in previous single centre studies of both doctors and students, the majority of students (74%) in our survey still wanted further education on antibiotic selection.

## Conclusion

Most final year students across seven European medical schools want further education on antibiotic selection, despite being at the end of their courses. Areas of non-confidence in prescribing were found, and comparing results with a similar survey of junior doctors, the students appear overly confident as to how effectively their current knowledge prepares them for being doctors. Educational programmes could benefit from including more cases of diagnostic uncertainty to guide students through the complexities of decisions in actual clinical practice, and from highlighting stewardship successes such as MRSA prevention as evidence for the importance of current interventions.

## Disclosure of interest

None declared.

